# A Mother’s Voice: The Construction of Maternal Identity Following Perinatal Loss

**DOI:** 10.1177/00302228231209769

**Published:** 2023-10-23

**Authors:** Larissa Rossen, Jessica E. Opie, Gypsy O’Dea

**Affiliations:** 1 Department of Counselling Psychology, 4402 Trinity Western University, Langley, BC, Canada; 2 The Bouverie Centre, School of Psychology & Public Health, 2080 La Trobe University, Melbourne, VIC, Australia; 3 School of Psychology, Centre for Social and Emotional Early Development, 2104 Deakin University, Geelong, VIC, Australia

**Keywords:** maternal identity, perinatal loss, mothers, motherhood, listening guide, qualitative method

## Abstract

**Background:**

Maternal identity, a mother’s internalized view of self as mother, has not been studied in relation to perinatal loss. This study aimed to investigate how women construct a sense of maternal identity after the loss of a baby.

**Methods:**

We interviewed 10 mothers who had experienced perinatal loss. A Listening Guide framework for narrative analysis was used to identify patterns of giving voice to the mother’s own story.

**Results:**

We identified 12 overarching voices which fell within three distinct groupings: voices of motherhood, voices of grief, and voices of growth. Although bereaved mothers grappled with constructing their maternal identity, they also demonstrated how maternal identity is individually and intuitively created through an honouring and remembering of the child that was lost, resulting in significant growth.

**Conclusions:**

There is need for a broader definition of what constitutes motherhood to encapsulate diverse mothering experiences, including perinatal loss.

## Introduction

Perinatal loss includes infertility during the preconception period, fetal death during pregnancy (miscarriage, ectopic pregnancy, induced abortion and stillbirth) and infant death in the first year of life (neonatal or post-neonatal death; Lawn et al., 2016; Public Health Agency of Canada (2020). The loss of a baby has profound, adverse emotional effects on bereaved parents (Burden et al., 2016; Huberty et al., 2016; Johnson & Langford, 2015), and is associated with severe anxiety, major depression, posttraumatic stress disorder, and suicidal ideation (Burden et al., 2016; Ellis et al., 2016; Heazell et al., 2016). Following a perinatal loss, for those who go on to have subsequently healthy pregnancies, there is typically increased health care use (Hutti et al., 2011) with higher rates of anxiety (Armstrong et al., 2009; Hutti et al., 2015) and postpartum depression (Armstrong, 2007; Blackmore et al., 2011). Prior research has considered grief responses, interventions, and lived experiences of mothers, fathers, healthcare professionals, and the wider family and community, in relation to perinatal loss. To date, the extant literature centres on parenting a living child following a perinatal loss, highlighting the connection between parenting identity with having a living child. Specifically, this literature has focussed predominantly on parenting pre-existing living children who were alive before the loss (Haylett, 2019), or on parenting subsequent children following loss (Campbell-Jackson et al., 2014; O’Leary & Warland, 2012; Warland et al., 2011). This emphasises the existence of a living child/ren as required for individuals to be acknowledged as parents.

Minimal research has focussed on the role of motherhood in relation to the infant that was lost. While a small body of literature identifies the importance of *continuing bonds,* a theory which emphasizes a continuing relationship with loved ones even after they have died, following perinatal loss (Field et al., 2013; Scholtes & Browne, 2015), it is important to highlight that *continuing bonds* is conceptually differs to maternal identity. Currently, motherhood and maternal identity are predominantly defined by physical aspects of caring for a child, such as nursing a baby and sustaining life (Hwang et al., 2022). When these practical dimensions of motherhood and material identify are removed, along with a baby, women are left with an identity of motherhood that is far from her expectations following a perinatal loss. Despite these women still being mothers, there is less sociocultural recognition of her identity as a mother and, hence, less supports are available for her mental and emotional well-being. Plagued with grief and distress, anecdotally, these mothers are left feeling confused and isolated, and may struggle to connect with or accept their identity of motherhood following the loss of their baby.

### Maternal Identity

Rubin (1984) introduced the concept of maternal identity into the nursing science literature to describe the psychological processes that occur during pregnancy and the postpartum period. Maternal identity is constructed during pregnancy, by way of an “idealized image of self as mother of this child” (Rubin, 1984, p. 39). During the postpartum period, maternal identity entails a shift in focus from an idealized image of the perceived self as mother to a view of the actual self as mother in relation to the child (Rubin, 1984). Throughout the process of getting to know their unborn infant during pregnancy and learning what to expect of their infants post-birth birth, maternal identity is progressively constructed, enhanced, consolidated, and reinforced (Walker et al., 1986). Mercer (1981) went as far as saying that maternal identity marks the endpoint of maternal role attainment. According to Rubin (1984), the core of maternal identity resides in the concepts “I” (mother) and “You” (infant), and the way these concepts influence each other. Therefore, maternal identity can be described as the personal and very specific relationship between a mother and her child (Walker et al., 1986). Despite the importance of this process, maternal identity has not been addressed in relation to women who have lost their babies in pregnancy and postpartum, possibly affecting the formation and acceptance of maternal identity and the subsequent well-being of bereaved mothers.

### The Current Study

In response to the limitations in the existing literature, this study aims to investigate the construction of maternal identity following the loss of a baby. This study will focus on maternal identity in relation to the child that was lost, to better understand motherhood following loss and motherhood more generally. The research will use a narrative framework of inquiry which gives voice to the mother’s story and own construction of maternal identity formation. This study aims to investigate how women construct a sense of maternal identity after the loss of a baby. The research question that guides this study is as follows: how do mothers form their identity as a mother subsequent to experiencing a perinatal loss?

## Methods

### Participants

Participants in this study were 10 mothers who had experienced a perinatal loss more than 5 years prior to participating in the study. We chose this timeframe to give women some time and space from the acuteness of the loss to be able to reflect and articulate the development of their maternal identity. This was an exploratory study which focused on depth of analysis using qualitative methods, hence, the sample size was small. Although the Listening Guide does not utilize the concept of ‘saturation,’ the researchers determined that no new voices were emerging after ten participants were recruited and analyzed.

To be eligible for study inclusion participants needed to be: (a) biological mothers of any age who lost their first infant to stillbirth, neonatal or infant death; (b) experienced the death of their biological child more than 5 years prior to participation in the research; (c) able to share about or identify with a maternal identity in relation to the child that was lost, and; (d) English proficient to participate in interviews. Participants were excluded if: (a) they had experienced a miscarriage as the primary loss, given this is a related but different type of loss in terms of length of time the mother had to construct her maternal identity, and; (b) who at the time of their baby’s death had a concurrent major loss, such as the death of a spouse or a parent. This decision was made as an additional bereavement may influence the grief process’ development and outcomes. Two participants completed consent forms but did not participate in either of the interviews of the study. No reason was provided for non-participation as the participants were uncontactable.

### Procedure

Ethics approval was granted by the Research Ethic Board of Trinity Western University (Ethics Approval Number: 21G05). Participants were recruited via word of mouth and social media outlets in North America and Australia. Recruitment occurred in a step-wise fashion. Interested participants contacted the first author and were subsequently briefed on the research and screened for the inclusion and exclusion criteria. If interested participants met eligibility criteria, consent was completed via email. Each mother was interviewed on two separate occasions to facilitate the development of a deeper relationship between the researcher and participant to generate richer disclosure. Both interviews were: semi-structured in nature, conducted via Zoom, recorded, and conducted by the first author (LR). Before the first and primary interview, participants completed a demographic survey, sent via email, to provide the researcher with a contextual frame for data interpretation. The primary interview invited each mother to share about how she constructed maternal identity after the loss of her first baby (mean length = 61 min, range = 52–82 min). During the follow-up interview (interview 2; approx. 30 min), referred to as a member check, the discussion centred on the mother’s experience of reading the analysis of her first interview as a means of ensuring the meanings created from the data analysis mapped onto her experiences. See Appendix A and B for the semi-structured interview schedule of interview 1 and 2, respectively.

Following data collection, all interviews were transcribed verbatim by LR, with some help by JO. All transcripts were deidentified throughout the transcription process. The transcription process comprised of listening to the audio recordings and writing verbatim everything verbalized by both the researcher and participant.

### Qualitative Data Analysis: The Listening Guide

A qualitative methodology was used. Qualitative research methodology is most appropriate when examining a new realm of inquiry and seeking to determine noteworthy issues (Corbin & Strauss, 2008), such as maternal identity in relation to perinatal loss. In particular, a Listening Guide (LG) feminist method for narrative analysis was utilized for this qualitative study (Gilligan et al., 2003). The LG is a qualitative, relational, voice-centered, and feminist methodology used to analyze interview transcripts. The LG emphasizes the psychological complexities of humans through attention to voice. Through the creation and special analysis of voice poems, LG attends to intonations in the interviews like silences, pauses, and non-verbal language. The methodological orientation and underpinning theory of this study is in line with the constructivist paradigm.

Members of the analysis team were trained in the LG method of analysis. The research team worked in pairs to conduct the multiple listenings (between 3–5 listenings) as required for the LG. Each analysis meeting focused on a different participant and lasted approximately 2-3 hours. The primary researcher (LR) was present for each participant analysis; There were a total of six additional researchers who contributed to the analysis including JO, GO, and four master of counselling psychology students.

The LG method of analysis comprises a series of sequential listenings (or steps), each designed to bring the researchers into relationship with a person’s distinct and multilayered voice by tuning in or listening to distinct aspects of the woman’s expression of her experience. In the present study, each listening involved reading of the participants transcript. Each listening required the active presence of the researcher and an acute desire to engage with the unique subjectivity of each research participant (Brown & Gilligan, 1992).

#### Step 1: Listening for the Plot

The first listening comprised two parts: (a) listening for the plot and (b) the listener’s response to the interview (Gilligan et al., 2003). The first listening was done by attending to what was happening and what stories were being told, as well as the landscape or multiple contexts within which these stories are embedded. In the second listening, the researcher focuses in on the voice of the “I” by following the use of this first-person pronoun.

#### Step 2: I-Poems

The second listening also includes constructing an “I-poem” by underlining the I-statements (pronoun and verb and other important accompanying words), then arranging the phrases like lines in a poem while maintaining the sequence in which they appear in the text.

#### Step 3: Listening for Contrapuntal Voices

The third listening, listening for contrapuntal voices, involved reading through the interview two or more times, then identifying and tuning into different aspects of the story and voices in the woman’s expression of her experience.

#### Step 4: Composing an Analysis

Having gone through each participants transcript a minimum of four times, an interpretation of the interview was developed from the data to synthesize the learnings of the participant’s experience in relation to the research question.

### Research Team

The research team comprised of three female researchers, all highly knowledgeable in perinatal research and experienced in qualitative methodologies. The first author (LR) is an Assistant Professor in Counselling Psychology, holds a PhD, is a Registered Clinical Counsellor in private practice specializing in perinatal loss and grief, identifies as female, and is a mom of three. She lost her first son, Brayden, who was stillborn at 38 + 3 weeks gestation. After her loss, she grappled to understand how she was still a mother without a child in her arms. She conducted the present research to understand how women can create and honour their identity as mothers and to support other bereaved parents. The research was conducted in memory and honour of her son, Brayden. The second author (JO) is a post-doctoral perinatal researcher, holds a PhD, and identifies as female. The third author (GO) is a Registered Psychologist specializing in perinatal mental health, holds a Doctor of Clinical Psychology, is a perinatal researcher, identifies as female, and is a mother to three children.

### Rigour and Quality

The following strategies were conducted to enhance the rigour and quality of this research’s data collections and analysis: (a) The primary researcher (LR) spent time building rapport with each participant to reflect the relational nature of this research and to facilitate the co-creation of meaning. All participants commented that LR’s loss helped them to feel safe and disclose their own personal stories of loss. Four of the participants in the study had a prior relationship with the primary researcher (LR) and were eager to participate to improve support for bereaved parents; (b) To achieve fairness, the deidentified transcripts and analyzes of these transcripts were shared with the analysis team; at least one team member in addition to the primary researcher were present for each analysis session to facilitate multiple perspectives; (c) Analyzes were initially completed by individual research team members before coming together to co-create and discuss final conclusions. All interpretations were shared amongst other members of the team and open for discussion if they felt the interpretations reflected any personal biases as opposed to the participant’s experience; (d) Follow up interviews, or member checks, with the participant were done consistently throughout the research process to facilitate ontological and catalytic authenticity. Participants provided correction and feedback on their individual analysis before the research team compiled the final analysis. Participants were also provided the final results before publication of the data, and; (e) All researchers kept field notes during and after the analysis process.

## Results

### Participant Characteristics

Mothers’ average age was 42 years (range: 32–47 years). Most women were married (*n* = 6), university educated (*n* = 8), worked full-time (*n* = 6), Canadian born (*n* = 7) and identified as Caucasian (*n* = 7). Women had between 0–3 living children, with an average of two losses (range: 1–5). Women who had experienced multiple losses were asked to focus on their first loss for the purposes of this study. Please refer to Table 1 for further, detailed participant demographics.

**Table 1.** Participants’ Demographics.

Participant Pseudonym	Age	Highest education	Current relational status	Occupational status	Country of birth	Country of residence	Ethnicity	No. and type of losses	Years since loss	Target loss for study purposes	No. of living children
#1 Serena	54	Bachelor’s degree	Married	Full-time	Canada	Canada	Caucasian	2 stillbirths (twins), 3 miscarriages	18 years	Stillbirth	0
#2 Darryle	47	Bachelor’s degree	Separated	Part-time	Canada	Canada	Filipino	1 stillbirth, 2 miscarriages	12 years	Stillbirth	2
#3 Vera	42	Master’s degree	Married	Part-time	Canada	Canada	Filipino	1 stillbirth, 2 miscarriages	9 years	Stillbirth	2
#4 Lauren	47	PhD	Married	Full-time	Germany	Canada	Caucasian	1 neonatal death, 1 stillbirth	10 years	Neonatal death	1
#5 Nora	32	College	Married	Full-time	Canada	Canada	Caucasian	1 stillbirth	6 years	Stillbirth	3
#6 Antoinette	36	College	Separated, defacto	Full-time	Canada	Canada	Caucasian	1 neonatal death	7 years	Neonatal death	1
#7 Jessica	42	Bachelor’s degree	Married	Full-time	Canada	Canada	Caucasian	1 stillbirth, 1 miscarriage	4 years	Stillbirth	3
#8 Sarah	44	Bachelor’s degree	Married	Full-time	Canada	Canada	Filipino	1 stillbirth	11 years	Stillbirth	3
#9 Alex	39	Bachelor’s degree	Separated	Part-time	England	Canada	Caucasian	1 stillbirth, 1 termination	6 years	Stillbirth	0
#10 Carly	41	Doctorate	Never married	Full-time	Australia	Australia	Caucasian	1 stillbirth, 3 miscarriages	3 years	Stillbirth	0

*Note.* Neonatal death = Death of baby within first 28 days of life; Stillbirth = Death of baby in the womb after 20 weeks of pregnancy; Miscarriage = Death of baby in the womb prior to 20 weeks of pregnancy.

### Voices

**Figure 1.**
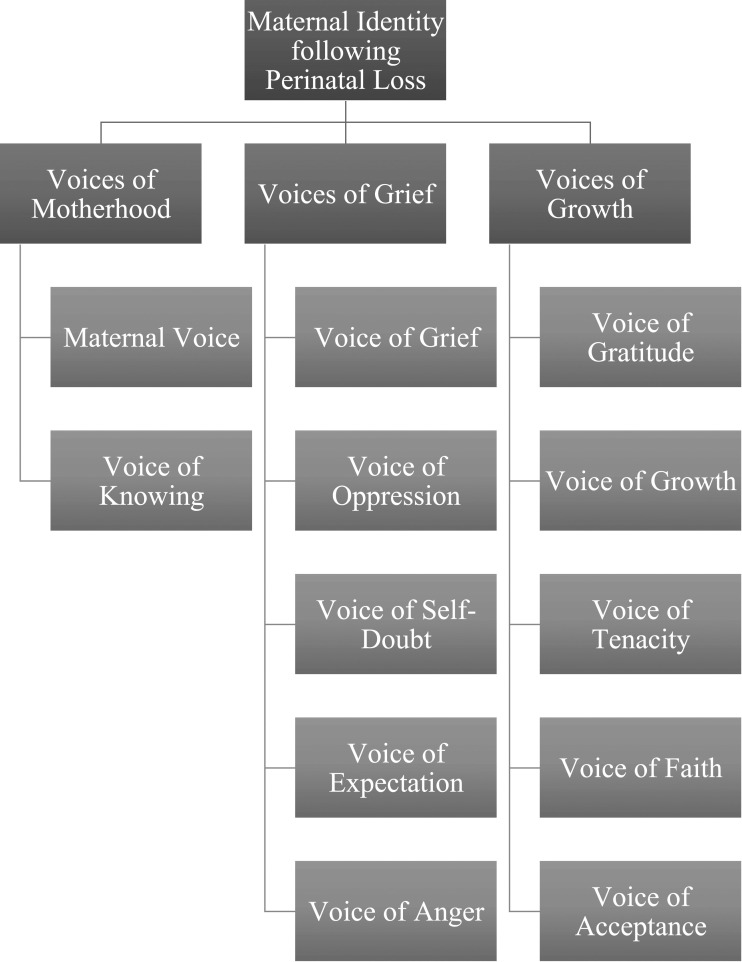
Voice Analysis.

The research team analyzed all interview transcripts according to the steps of the LG and identified a total of 12 overarching voices. These voices largely represented the participants’ stance toward constructing a sense of maternal identity following the loss of their child. All voices fell within three broader and distinct groupings: voices of motherhood, voices of grief, and voices of growth (see Table 2 and Figure 1). These voices will be outlined in the following section.

**Table 2.** Voice Analysis.

Voices of motherhood	(*n*^a^)	Participant #	Voices of grief	(*n*)	Participant #	Voices of growth	(*n*)	Participant #
Maternal voice	10	All	Voice of grief	10	All	Voice of gratitude	8	1, 3, 4, 5, 6, 7, 8, 10
Voice of knowing	4	2, 3, 4, 7	Voice of oppression	5	1, 2, 4, 5, 10	Voice of growth	7	1, 2, 3, 5, 7, 9, 10
			Voice of self-doubt	5	1, 2, 4, 6, 7	Voice of tenacity	5	1, 2, 3, 9, 10
			Voice of expectation	5	2, 3, 6, 9, 10	Voice of faith	3	2, 7, 8
			Voice of anger	4	1, 2, 8, 9	Voice of acceptance	2	4, 8

^a^Number of participants who spoke using this voice. Please note that each participant had multiple voices identified.

#### Voices of Motherhood

##### Maternal Voice

The maternal voice was present and dominant in all 10 mother’s narratives and was characterized by a strong connection to and yearning to be with their children. The most prominent aspect of the maternal voice was that it was strongly interconnected and intertwined with grief. Mothers oscillated between feeling deeply connected to their lost child and feeling moments of deep grief. Serena (Participant #1) described this as a yearning to be together with her child, while Carly (Participant #10) described an emotional depth and protectiveness that she has come to associate with motherhood, wholly enmeshed in her connection to her daughter. Antoinette (Participant #6), on the other hand, described how her maternal identity was seated in sadness, reporting that she had made very few happy memories with her stillborn son, since there was so much grief and sadness surrounding him.

The maternal voice was also noted in how mothers honoured and remembered their children. Serena (Participant #1) described the ways she celebrated her lost baby’s legacy through creating a garden, lighting candles, planting trees, talking to her children, and feeling their presence. Serena described a connection to her babies that she wanted to last forever: “In the hospital we were given their footprints and I went and got them done in bronze because I wanted everything to last forever… I wanted everything about them to last way longer than me.”

The maternal voice was also reflected in a strong need for the mothers to be recognized as mothers, in the absence of their child. Jessica (Participant #7), for example, was confronted with questions of whether she believed herself to even be a mother, and her inner maternal voice fought through the doubts about her motherhood identity and rose to the surface as she concluded, “I’m still a mom … I don’t need to have a physical child to be a mother.” Carly (Participant #10) shared: “you’ll forever be a mom because you now understand the depth of feeling that comes from being a mom.” The maternal voice appeared as Carly described fighting to give her daughter a chance at life when the doctor told her there was little hope. This protectiveness continued as she fought to maintain a legacy for her daughter, through advocacy and living her own life to the fullest.

Lastly, the maternal voice is also reflected in some mother’s connecting and supporting other bereaved parents. Vera’s (Participant #3) view of motherhood expanded as she described her sense of maternal identity being connected to supporting other mothers. She said:

You know that that’s actually a big part of motherhood is making sure that you're around other mothers to, you know, loss or not, to be around and to support each other and I guess I kind of viewed motherhood before maybe too insular and at least now I’ve kind of felt like it shouldn't be that way.

##### Voice of Knowing

The voice of knowing was identified in four participant stories and was reflected by a sense of self-assuredness that became apparent when other stronger voices were stripped away. The voice of knowing emerged as a strong initial desire to want to be a mother. For example, Vera (Participant #3) always knew she wanted to be a mother and pursued a career that was conducive to family life. The voice of knowing was also recognized by an inner intuition the mothers possessed during the loss of their children. Darryle (Participant #2) had a strong sense of knowing which came through in the following quote: “I knew him, there wasn’t anything I could do, and I knew that the erratic movements were not normal.”

#### Voices of Grief

##### Voice of Grief

The voice of grief was strongly present in each mother’s story and carried a distinct tone of hopelessness and despair as mothers in the study described wrestling with their new mothering reality after losing their child. In her own words, Serena (Participant #1) said “you’re so empty and you have a broken heart, a physically broken heart over the loss of your children.” Serena discussed unseen grief, unacknowledged grief, in-your-face grief, private grief, comparative grief, and societal stigma about grief. Despair was also present as Darryle (Participant #2) recounted what she considered the deepest pain a parent can go through, the loss of a child: “I questioned my purpose in life, like God where do you want me to go from here?” Nora described the feeling of having a hole in her heart, but also a hole in the family structure as well, while Antoinette (Participant #6) identified the challenges of parenting amidst her grief, highlighting the intersection between the voice of grief and the maternal voice.

One of the most prominent intersections of the maternal voice and grief voice was Jessica’s (Participant #7) expression that she “failed” as a mother and/or to become a mother in the first place. This sentiment highlighted that her grief was enmeshed in the construction of her maternal identity; the two could not be separated, as grief was present when she was in the process of identity construction. Lauren (Participant #4) poignantly recounted that she not only felt robbed of motherhood, but she felt shut out by society because no one knew how to how to handle it, how to talk to her. The voice of grief highlighted the notion that bereaved mothers often did not feel supported by friends or family. Many of their friends had not experienced similar losses which caused distance in the mother’s ability to relate to them. Mothers in the study felt a longing to be grounded and supported by family members; however, at times, these family members were affected by their own unprocessed grief and trauma and, in turn, were unable to give the care and love those mothers needed.

##### Voice of Oppression

The voice of oppression was present in half of the women’s stories and was heard when mothers spoke of the barriers and invalidation to forming their maternal identity, and the silence and oppression they have experienced by society more generally following the loss of their children. The voice of oppression also represented a form of self-silencing due to society’s response to grief and the death of a baby before, during, and after birth. For example, Serena (Participant #1) recounted how she felt society made it difficult for her to really identify as a mother because of the length of time her children existed while Nora (Participant #5) recounted the difficulty she faced when others asked about how many children she had. Mothers also spoke about the difficulty of dealing with societal norms such as Mother’s Day, Father’s Day, and Christmas, and their experience of others avoiding talking about, or acknowledging, their babies, their loss, and their grief. The voice of oppression was also recognized as mothers recounted the language, terminology, and medical terms that are used to describe the loss of a baby. Darryle (Participant #2) described in her own words: “And that just adds salt to the wounds of a mother who is looking at the report and going well, what are you grieving for? This is just fetal demise or this is a spontaneous abortion.” The voice of oppression was, therefore, imposed upon mothers by the discomfort of others, placing them in a position to defend themselves as mothers. Carly’s (Participant #10) words perfectly encapsulated this voice:

I think it’s the idea of how in a world that is so governed by language that a word like “mother” doesn’t encapsulate an experience of loss… So, the meaning that we’ve created around the word ‘mom’ it’s probably not very reflective of everyone’s experience.

##### Voice of Self-Doubt

The voice of self-doubt was present in five participant stories, and was recognized by tones of regret and guilt, particularly in relation to the death of their children. Lauren (Participant #4) said: “If anything happens to your child, every mom somehow feels responsibility… kind of a guilt, is my body even able to have living children?” Darryle (Participant #2) questioned her choice to stay home and pursue a natural birth when she was pregnant with her son rather than listen to her motherly instincts that something was wrong. She recounted in the following quote: “Could I have followed my prompting… I knew that something was wrong and I listened to those people that said don’t go to the hospital. Would he still would he be here in my arms at this time?”

##### Voice of Expectation

The voice of expectation was recognized in five participant narratives, and was marked by tones of hope, perspective, and belief about what motherhood might entail. This voice was reflective of normative scripts around motherhood that provide women with expectations for the course of birth and parenting; scripts that left them without language for their experience when expectations were not met. Vera (Participant #3) never expected to experience loss or struggle in her journey to motherhood while Alex (Participant #9) felt she should have been more prepared or had a plan for the possibility of a stillbirth.

##### Voice of Anger

The voice of anger was illustrated in four participant stories and was recognized by a frustration and spitefulness generally directed towards other people in the early stages of grief after their loss(es). For example, Alex (Participant #9) stated, “when I saw pregnant women, I hated them. Not because they were going to have a baby. I hated the innocence.” Alex explained that these feelings were part of her grieving process, as she projected her own experience of loss onto other expecting mothers. Serena (Participant #1) described having her “bats out all the time” after the loss of her twins and said, “I was just fighting everybody, and I just didn’t care.”

#### Voices of Growth

##### Voice of Gratitude

The voice of gratitude was present in eight participant stories and was recognized by tones of thankfulness and appreciation. The voice of gratitude was highlighted in Nora’s (Participant #5) story in relation to the way that her stillborn son was honoured and cared for by nurses when he died. She said that the attending nurse was “talking to him and cooing to him and just being so gentle with his body” which made Nora feel like she was really honouring them (as parents) at that time. Several women spoke of their gratitude towards their partners. For example, Lauren (Participant #4) and her partner leaned on each other and honoured one another as parents, which was an important part of Lauren’s maternal identity construction. Vera (Participant #3) and Nora (Participant #5) tell of the longevity of gratitude that they experienced in creating strong, authentic friendships within the bereavement community.

##### Voice of Growth

The voice of growth was found in seven of the participant narratives, and was marked by tones of openness, experience, reflection, wisdom, and perspective, gained after the loss of their children. Nora (Participant #5) described the revelation that feelings are more complicated after loss - that “you can feel both (joy and sadness) at the same time.” Nora’s newfound sense of valuing the present moment in the following quote demonstrated the wisdom she has gained: “I understand how quickly it can all go away.” The voice of growth for Alex (Participant #9) expressed the total transformation that occurred after the loss of her daughter, helping her to be her more attuned to the pain of others. Carly’s (Participant #10) voice of growth is evident in her renewed ability to encounter others in their suffering, inviting others to acknowledge their grief as a part of the human experience, and creating space for collective healing.

##### Voice of Tenacity

A tenacious, persistent, and driven voice was present in five of the mothers’ stories, and was recognized by a fighting spirit and perseverance, particularly related to the journey of conception and motherhood, and maternal identity construction. For Alex (Participant #9), the voice of tenacity was an action-oriented voice that motivated her to prove that she was a mother, to prove that she could have another baby, and to improve the birthing curriculum to adequately prepare expecting mothers for stillbirth experiences. For Carly (Participant #10), the voice of tenacity presented as a resistance to being defined by her loss. Carly began a process of reconstruction, to move beyond her grief for herself and her daughter. She stated, “I want [my daughter] to be proud that I would have been a mom… so I’m going to make sure that my life is rich and honours both of us.”

##### Voice of Faith

The voice of faith presented itself in three participant narratives, and was recognized by tones of purpose and meaning, particularly as mothers processed and found meaning, healing, and their sense of maternal identity following the loss of their babies. Jessica (Participant #7) described this experience through the birth of her daughter as a “tremendous burst of light and energy and joy,” as she described her daughter “transitioning to her angel.” Sarah (Participant #8) spoke about the ability to communicate with her child through prayer, and the belief that their reunion would come to fruition in the afterlife.

##### Voice of Acceptance

The voice of acceptance was found in two participant stories and was recognized when mothers spoke with clarity, self-assuredness, and a sense of peace in relation to the loss of their children. This voice was also associated with a letting go of anger. For Sarah (Participant #8), the voice of acceptance was rooted in her assurance that her son was delivered to heaven through the grace of God and highlighted in the following: “I think God’s grace just said, this what I’ve chosen for you. I just need you to accept it. And I don’t like it, but I accepted it.”

## Discussion

This research aimed to investigate women’s construction of maternal identity following perinatal loss. The findings, which followed a Listening Guide methodology, revealed 12 overarching voices which fell within three broader and distinct groupings: voices of motherhood, voices of grief, and voices of growth. The voices of motherhood overlapped with the voices of grief as mothers grappled with constructing a motherhood identity following the loss of their baby, primarily due to the stigma and silence associated with child loss. The mothers in this study also demonstrated how maternal identity is individually and intuitively created through an honouring and remembering of the child that was lost. As a result, the voices of growth emerged as these women experienced significant development following their loss as they honoured their own identity as mothers. The study corroborates the extant literature which demonstrates the deep pain of perinatal loss and the significant, long-lasting impacts that mothers experience on their journey to motherhood, and the way they identify as mothers. The novelty of this study shows that, even after the death of a child, mothers still develop a very personal and specific relationship with their child and that, after the loss, the construction of maternal identity, in the traditional sense, appears to be disrupted in many ways.

### Maternal Identity and Grief Following Perinatal Loss

In the present study, mothers grappled with a complex interplay of feelings that are associated with balancing the expectations of what one felt motherhood would entail with the overwhelm and confusion surrounding being a mother to a child that has died. Grief emerged as a central theme in participant stories of motherhood and how they defined and constructed a sense of maternal identity following loss. Following perinatal loss, research has uncovered various forms of grief such as ambiguity and disenfranchised grief (Lang et al., 2011), complicated grief (Kersting & Wagner, 2022), and factors affecting grief (Kishimoto et al., 2021), that bereaved parents face in their interactions with friends, family, society, and healthcare professionals. Doka’s (1989) concept of disenfranchised grief is used to describe the experience of loss, or a state of bereavement, not openly acknowledged, publicly mourned, or socially supported. This study adds to the research by elucidating how mothers construct their sense of maternal identity, and how grief moved from deep sadness and pain into gratitude and honouring of those children that have been lost too soon. Mothers in this study showed the importance of recognizing grief and moving towards giving it the space that it needs, while at the same time holding openness for the joy and love mothers feel towards their children.

### Sociocultural Constraints to Maternal Identify Following Perinatal Loss

The study also highlighted, from mothers’ own stories, the broader expectations, silencing, and oppression that occur after the loss of a baby which make it difficult for women to understand and construct their sense of maternal identity. Layne (1997, p. 291) writes that pregnancy losses are subject to “the triple edict of modern puritanism– ‘taboo, nonexistence, and silence’.” According to De Vault (1999, p. 177) silencing can refer to “quieting... censorship, suppression, marginalization, trivialization, exclusion, ghettoization, and other forms of discounting,” which women in this study described at length. Women described silencing not only in terms of speaking, but also in terms of not being acknowledged, not being present, not being heard, being ignored, and not being seen. These silencing practices are embedded in day-to-day relationships - the silences and the silencing words of colleagues, friends, and family members and the self-imposed silences of women who experience such losses. Silencing has been explored in the context of workplace and organizational silencing of bereaved parents returning to work after loss (Meunier et al., 2021). Pollock and colleagues (2020a, 2020b) explored qualitatively bereaved parents experiences of ‘stillbirth stigma’ which was reported by ∼38% of participants (*n* = 817) and included shame, blame, devaluation of motherhood, and discrimination. Our study adds to the literature in highlighting how societal and self-silencing affect women’s construction of maternal identity following loss. Mothers in this study showed the struggle they faced in feeling robbed of motherhood and being part of a secret ‘maternal loss society’. The silence mothers experienced was often cited in relation to an ignorance towards and discomfort around grief in our society. Women also collectively identified a lack of language and representation for experiences of loss in the definition and understanding of motherhood. Yet, most importantly, our study demonstrated the courage and bravery mothers displayed in mothering their deceased children. Women in this study fought hard to recognize and honour their motherhood, often while fighting to conceive their children or simultaneously supporting other women devastated by loss. Despite the silence, women kept hold of a special, internal place close to their hearts where they acknowledged their motherhood, privately and with those whom they felt close.

### Maternal Identity and Growth Following Perinatal Loss

The current study also highlighted instrumental growth that is part of maternal identity construction following the loss of a baby. The mothers in this study demonstrated how maternal identity is individually and intuitively created through an honouring and remembering of their lost child’s legacy. In this way, women created meaning and purpose out of their loss and in doing so created a sense of maternal identity as a way of respecting their children. An emerging yet scant literature has examined aspects of growth following loss such as post-traumatic growth (Cacciatore et al., 2018; Ryninks et al., 2022), hope (de Andrade Alvarenga et al., 2021), parental empowerment (Murphy, 2012), unsilencing (Gillis et al., 2020), and continuing bonds (Jones et al., 2021). Our study corroborates these results and further adds to these experiences in the context of maternal identity attainment. The mothers in our study showed us how to find meaning and generosity amidst painful experiences that each of us encounter in our lives. These mothers taught us the importance of how individuals can transform their story of loss and despair into a meaningful narrative of honour and recognition which can ripple into the lives of other families and the community, to honour their identity as “mother”. The mothers in this study taught us that the fragility of life is to be appreciated in every moment, and the importance of perseverance, community, and connection in healing and developing a sense of maternal identity following perinatal loss.

### Strengths and Limitations

While the current research provides meaningful insight into the maternal identity of mothers following perinatal loss, the following strengths and limitations of the study have been identified. One of the key strengths of the study is the richness and depth of women’s experiences that was gained, in their own words, in relation to how they identified with being a mother following the loss of their baby. By implementing the LG approach, our findings unpacked the mothers’ multilayered voices and provided deeper understanding of women’s experiences of grief and gratitude, pain and perseverance, oppression and acceptance, self-doubt and faith, as they navigated maternal identity post-loss, as perceived by the mothers themselves. It might be considered a limitation that women included in the study were those who were able to vocalize their experiences of maternal identity following the loss of their baby. The research may not have captured the experiences of women who may find it difficult or have yet to acknowledge their maternal identity following loss, due to silence, oppression, or other factors that were not explored in the present research. Further, the sample of participants represented a cohort of women who were relatively well educated, and homogenous in ethnicity and socioeconomic status.

### Implications

#### Theoretical

In Western society, the concept of motherhood is equated with the biological processes of pregnancy, birth, and lactation, which are assumed to produce a special maternal bond between mother and child (Park, 2013). When mothers lose a child during pregnancy and/or postpartum, these activities and responsibilities associated with motherhood are removed, resulting in bereaved mothers struggling to identify with motherhood in the traditional sense. In our study, all of the mothers naturally identified with being a mother, however found the narrow social construction of motherhood created an internal schism or tension which left participants feeling doubt, confusion, and disconnection with their identity as mothers. Some mothers described a discord between an outward display of motherhood and inner identification of being a mother. Overall, mothers found the invalidation and silence created barriers to forming their maternal identity following the loss of their children. One mother encapsulated this tension well in the following question: “I mean how do you parent a dead child?” This sentiment expressed how mothers try to mould their experiences of motherhood into the broader societal understanding of the concept, which does not fit or make sense in the case of loss.

Therefore, this study highlights the need for a broader definition and understanding of motherhood, which encapsulates diverse experiences of motherhood and mothering. Rich (1981), p. xiii) oft-cited quote “We know more about the air we breathe, the seas we travel, than about the nature and meaning of motherhood” captures the need for academic disciplines, from anthropology to women’s studies that are engaged in some form of motherhood research, to continue imploring the need to learn more about the nature and meaning of motherhood beyond biological processes. Concurrently, there is also a strong need for addressing the taboo and silence surrounding the death of child, especially an infant loss during pregnancy and postpartum. Horton and Samarasekra (2016, p. 515) contend that ‘perhaps the greatest obstacle to addressing stillbirths is stigma.’ Pollock and colleagues (2020a, 2020b) have called for ‘stillbirth stigma’ to be reduced overall, with an important aspect of this being women’s identities. The present research echoes this call in addressing the societal stigma surrounding perinatal loss and motherhood. As a collective, we can challenge the status quo, yet research has often ignored the growth and empowerment that can come from child loss (Murphy, 2012). Our research highlights the agency and tenacity that bereaved mothers possess in bringing about change while simultaneously honouring their children and forming their maternal identities. As a collective, we can be part of honouring, remembering, and granting purpose and meaning to mothers. The responsibility for collective change is not only at the hands of bereaved parents, but the allyship of multiple disciplines which target multiple levels within our society. Collective change requires collective action.

#### Clinical

Clinicians have an important role to play in breaking the silence present in perinatal loss and in supporting women construct their maternal identity following the death of a child. The most helpful aspects of counselling for bereaved parents have been shown to be psychoeducation regarding emotions and emotional regulation, the normalization of intense emotions, and simply having a supportive and nonjudgmental clinician with whom to discuss their pregnancy loss (Bennett et al., 2012; Brier, 2004). Support groups have also been demonstrated to be helpful in supporting mothers and their maternal identity development post loss (Bennett et al., 2012; Gold et al., 2012). Clinicians can address and validate the various, complex, and, at times, confusing voices that emerged in this study, including the maternal and knowing voice, voices of grief, and voices of growth. Specific to the voices of grief, therapists can help recognize and explore these within the therapy relationship. The voice of invalidation, expectation, anger, and self-doubt are especially important to name and to offer the kind of support and validation to the mother that is so often denied in relationships outside of therapy.

Rituals and traditions can be helpful in bringing the mother’s grief out of isolation and into a safe space, wherein others are available for support, validation, and comfort. In contrast to other types of losses, there are no communal rituals for grieving a perinatal loss. For example, there are no customary religious or social gatherings, no condolence cards or flowers, nor is there even a death certificate (Markin & Zilcha-Mano, 2018). In fact, perinatal loss is the only type of loss in Western society where there are no culturally sanctioned rituals or traditions to help the bereaved to say good-bye (Markin & Zilcha-Mano, 2018). Therefore, therapists have a unique opportunity to suggest and create personally meaningful mourning rituals and traditions that are relevant to bereaved parents. Examples include rituals like creating a grave site and writing letters to the lost baby, which may help the parent to grieve.

#### Research

From a research perspective, there are various avenues for further exploration that arise from this study. Research pertaining to fathers and parenting identity would be a logical follow-up from this study. The construction of sibling identity relative to perinatal loss could also be addressed for those who have lost a sibling. Further mixed method or quantitative research developing and/or utilizing an adapted maternal identity scale might also help us further understand the constructs underlying the maternal identity of mothers following perinatal loss. The relationship between the experience of stillbirth and social constructs, gender, parenting, stigma and attachment also requires closer attention. Further research on this topic from a clinical/counselling perspective is clearly needed.

## Conclusion

This study investigated the construction of maternal identity following the loss of a baby and found three groups of voices: voices of motherhood, voices of grief, and voices of growth. Mothers in the study were deeply connected to motherhood but often grappled with grief and invalidation in navigating their identity as mothers. Despite the pain, mothers in our study revealed how they found meaning, gratitude, and growth through their journey of loss and motherhood. These mothers teach us about transforming loss and despair into honour and recognition of a child, which ripples into the lives of other families and the communities. We hope this research breaks the silence, shame, judgement, confusion, and misinformation that surrounds pregnancy and infant loss and helps to equip clinicians, researchers, mothers, and families to navigate loss and motherhood following the death of a baby.

## Supplemental Material

Supplemental Material - A Mother’s Voice: The Construction of Maternal Identity Following Perinatal Loss Supplemental Material for A Mother’s Voice: The Construction of Maternal Identity Following Perinatal Loss by Larissa Rossen, Jessica E. Opie, and Gypsy O’Dea in OMEGA - Journal of Death and Dying
