# Supplementary material for: A Mother’s Voice: The Construction of Maternal Identity Following Perinatal Loss
Source: Omega (Westport). 2023 Oct 23;92(3):1559–81. doi: 10.1177/00302228231209769 (PMC12769918; doi:10.1177/00302228231209769)
Supplement: Supplemental Material - A Mother’s Voice: The Construction of Maternal Identity Following Perinatal Loss [file sj-pdf-1-ome-10.1177_00302228231209769.pdf]

Appendices

Appendix A

First Interview Guide

Participant #: **Interviewer Name:**

Date: **Interview Start Time:**

Introductory Script: *Thank you for choosing to participate in this interview. Before we proceed, I want to remind you that, at all times during the interview, you may choose to disclose or not to disclose any information, depending on how comfortable you feel. You also may request to take a break or to discontinue the interview at any time.*

1. <u>Contextual Component</u>
   Preamble: As you know, we are interested in women's experiences of motherhood following the loss of a baby. This is the first of two interviews, and the purpose is to collect information about what you have experienced and how it has affected you.

   - As a way of getting started, perhaps you could tell me a little bit about what it means to be a mother to you?
   - Can you tell me a little about the loss of your baby (query for baby's name, what time point in pregnancy the baby was lost, whether there was a cause of death)

2. <u>Narrative Component</u>
   I'm now going to ask you to please describe for us in as much detail as you can remember your experiences of motherhood following the loss of your baby.
   In this experience of loss, what most shaped your identity as a mother?
   Is there anything that made it more difficult for you to embrace your identity as a mother?
   Has the way you see yourself as a mother shifted over the years (query: what has consolidated/helping and interrupted/hindering) positive, negative, helping, hindering)? What have these shifts been connected to?

   Summarize what has been discussed up to this point with the participant and offer any further reflection or changes, as necessary.

3. **<u>Demographics Component</u>**

   i. Age

   ii. Education

   iii. Marital Status

iv. Parental Status (note: single parent, guardianship/custody arrangements)

v. Occupational Status (note: employed F/T, P/T, on leave, etc.)

vi. Income level (household)

vii. Country of birth
- ❑ If not Canada, (a) length of time in Canada; and (b) 1st language

viii. Ethnic and Cultural Identification: ___________________________________
Query for any culture-specific perinatal practices (e.g., traditional Chinese confinement period):
____
____
____

ix. Date of child's birth: ___________________________; Calculate _____ years _____ since child's death.

x. Type of Perinatal Care Provider (GP, Midwife, OB): ___________________

xi. Number (Total:______) and Dates (year, list below) of Live Births:
____
____
____
____
____

xii. Number (Total: ______) and Dates (year, list below) of Previous Miscarriages, Still Births, other Perinatal Losses (note type below):
____
____
____
____
____

xiii. Children's Information (ages, if with current partner?, and note if adopted)
Age: Child with Current Partner? YES□ NO□ Adopted? YES□ NO□
Age: Child with Current Partner? YES□ NO□ Adopted? YES□ NO□
Age: Child with Current Partner? YES□ NO□ Adopted? YES□ NO□
Age: Child with Current Partner? YES□ NO□ Adopted? YES□ NO□
Age: Child with Current Partner? YES□ NO□ Adopted? YES□ NO□

Interview End Time: ________________ **Length of interview:** ________________
Appendix B

Second Interview Guide

Participant #: **Interviewer Name:**

Date: **Interview Start Time:**

Introductory Script: *At this point, I would like to provide you with information about what happens next during this research process. I have created a written summary of our first conversation together. I am providing a copy of this summary for you to review before our next meeting. I will be reflecting on the summary, noting down any questions, thoughts or comments that I have from my reading. I hope that you will do the same.*

We will then meet to discuss the summary, for the purpose of collaboratively developing a narrative or story about your experiences, which I will then use for my final analysis. If you have any questions in between our conversation today and the next time we meet, please don't hesitate to call, 778-513-0133.

Second Interview Protocol
The following questions are just illustrative of the kinds of questions that may be asked during the interview.

1. What was it like to read through the transcript?
Emotional, reliving it, husband read it, interesting
   a. What was your initial reaction to your story? How did you react upon rereading your story (if you did so)?
   b. In what ways did the text reflect how you understand yourself as a mother?
   c. In what ways did the text reflect a shift in how you understand yourself as a mother? Was there anything that you discovered or become aware of during the interview that is reflected in the text?
   d. Where does the text need to be explained or altered as a result of the fact that you might feel as though you were misunderstood?

2. What was it like to share about your experience of motherhood following the loss of your baby (i.e. emotionally, spiritually etc)?
   a. When have you shared this with another person before?
   b. When you shared this with another person, did you do so readily? Why or why not?
   c. In the past have you explained your experience of motherhood in a different way?
   d. If you have explained your experience in a different way, why did you feel as though you had to explain your story in that different way? What or who influenced you to tell your story in that different way?
   e. When did you notice that you began to tell your story or understand your experience in a different way? What or who influenced you to tell your story in this new way?
